# Influence of Functional Movement, Jumping Ability and Linear Speed on Change of Direction Speed in Female Basketball Players: Novel vs. Traditional Approaches

**DOI:** 10.5114/jhk/196780

**Published:** 2025-04-30

**Authors:** Francisco J. Barrera-Domínguez, Paul A. Jones, Bartolomé J. Almagro, Jorge Molina-López

**Affiliations:** 1 Faculty of Education, Psychology and Sport Sciences, COIDESO, University of Huelva, Huelva, Spain.; 2 Directorate of Sport, Exercise and Physiotherapy, University of Salford, Greater Manchester, United Kingdom.

**Keywords:** agility, multidirectional speed, key performance indicator, team sport, performance

## Abstract

There is extensive discourse surrounding the factors influencing performance in change of direction (COD) actions in basketball, given the wide range of tests and methods that exist in the scientific literature for assessing this ability. This study analysed and compared relationships between functional movement, jumping and linear speed performance using two distinct methods of measuring COD performance in female basketball players, while also distinguishing between cut-off angles. Fifty female semi-professional basketball players (age: 23.7 ± 3.81 years, body height: 175.5 ± 7.69 cm; body mass: 64.4 ± 7.88 kg) participated in the assessment, involving various performance and COD tests. COD tests were categorised for each method and angulation into “low performance” and “high performance” groups, facilitating a Bayesian comparative analysis. For the traditional method of measurement (execution time) vertical (ES ≥ 0.66; BF_10_ ≥ 3.50) and horizontal (ES ≥ 0.97; BF_10_ ≥ 44.4) variables exhibited significant differences between performance groups across all cutting angles, with faster players performing better in all tests. For the novel approach (COD Deficit) differences between performance groups were only found for horizontal variables, while these differences disappeared for vertical variables. These findings suggest the need for caution when considering the determinants of COD performance, as these relationships are directly dependent on the COD method used. Therefore, female basketball coaches are encouraged to adopt the COD Deficit for assessing this ability, as it isolates COD from other abilities.

## Introduction

Changes of direction (COD) and speed constitute actions that recur more than a thousand times during a women’s basketball game (Matthew and Delextrat, 2009), responding to the game’s specific demands (Sekulic et al., 2017). In basketball, 15.1% of COD actions are executed at maximum intensities (<−3.5 m•s^−2^) (Svilar et al., 2018). These high-intensity movements play a pivotal role in determining the final outcome of the match, given the fast-paced nature of the game and the brief duration of the decisive actions in both offensive and defensive scenarios. Consequently, enhancing COD abilities is deemed essential for gaining a physical edge over opponents in basketball (Brini et al., 2020), irrespective of the playing position and gender (Power et al., 2022). In addition, throughout a basketball game, COD are executed at a multitude of different angles, spanning from 0° to 180° (Gonzalo-Skok et al., 2023). For all these reasons, basketball coaches must be cognizant of the critical requirements for improving this skill and fostering the multidirectional speed development of their players.

COD represents a complex and multi-directional action, defined as the ability to decelerate and accelerate in a planned manner toward a new direction (Nimphius et al., 2018). Despite existing scientific literature highlighting the significance of ankle dorsiflexion, dynamic balance, linear speed, jumping ability, and the COD technique itself as determinants of COD performance (Barrera-Domínguez et al., 2020; Chaouachi et al., 2009; Hewit et al., 2013), a consensus remains elusive, leading to considerable controversy regarding which factors truly determine COD performance. In this regard, although prior studies with male basketball players have identified a relationship between quantitative movement tests, such as ankle dorsiflexion and dynamic balance, with COD (Gonzalo-Skok et al., 2015), others have failed to observe such a relationship (Barrera-Domínguez et al., 2020). Regarding speed, studies involving female basketball players demonstrate a robust association between linear speed and COD actions (Michael et al., 2021). However, conflicting findings exist, with some studies not observing this correlation (Nimphius et al., 2013, 2016). In terms of jumping, previous research suggests that plyometric exercises with a short stretch-shortening cycle are generally the most specific to COD actions (Falch et al., 2020a). Nevertheless, this relationship may not occur in female basketball players, where long stretch-shortening cycle and strength exercises might be more specific to COD performance (Barrera-Domínguez et al., 2024a).

The discrepancies in previous studies could be explained in different ways. On the one hand, the utilization of more than 48 different COD tests in studies with basketball players has been noted (Sugiyama et al., 2021). Specific characteristics of the different COD tests (i.e., approach distances, angulations, and numbers of cuts) may lead to different magnitudes of physical and technical requirements for each test (Nimphius et al., 2018), introducing a potential limitation when comparing data between studies. As COD performance is directly dependent on the cutting angle (Dos’Santos et al., 2018), the use of tests with different cutting angles directly affects the performance and test-influencing variables, adding complexity to data interpretation and comparison (Falch et al., 2020b; Nimphius et al., 2018; Skalski et al., 2024). Consequently, it is not advisable to compare results between studies employing different COD tests. To enhance standardization, it is strongly recommended to employ several COD tests with a single cut at different angles and at the shortest possible approach distance (at least 5 m), aiming to minimise the influence of other physical qualities on the test and to create a “COD angle profile” of each player (Gonzalo-Skok et al., 2023; Nimphius et al., 2016). On the other hand, two distinct methods are currently employed to measure performance in COD tests. The traditional approach involves assessing total time or average velocity in the COD test, while the novel method, known as the change of direction deficit (CODD), seeks to quantify the time an athlete spends in the cutting action itself. CODD expresses the difference between the time it takes to complete a COD test and the time taken to cover the same distance in a straight line as a percentage. These data provide insight into the athlete’s efficiency in executing COD actions relative to their linear speed (Freitas et al., 2021b). The use of total time or average velocity in the test may encompass other physical capabilities influencing the final result, such as linear speed, anaerobic capacity, and movement specificity for the test (Nimphius et al., 2013, 2016). In this sense, previous studies indicate that linear speed can contribute to as much as 74.8% of the total time in a COD test (Delextrat et al., 2017). Consequently, CODD has been proposed as a potentially more valid variable for evaluating COD performance (Freitas et al., 2021b; Nimphius et al., 2013, 2016), as it accurately reflects the targeted physical quality (i.e., COD performance), eliminating the influence of other physical qualities on the test result.

Women’s competitive sport has been on the rise in recent years, which contrasts with the current underrepresentation of women in research within sport and exercise sciences (Anderson et al., 2023). Therefore, observing the lack of consensus in the scientific literature (Michael et al., 2021; Nimphius et al., 2013, 2016) regarding the most determining physical factors in COD actions for female basketball players, a study is needed that analyses the relationship between the different performance variables and COD performance using both prevalent methods for evaluating performance in these actions, total test time and CODD. Therefore, the present study aimed to analyse and compare the relationships among functional movement, jump and linear speed performance using two different methods of measuring COD performance in female basketball players, with a focus on differentiating between cut-off angles. It was hypothesized that measuring COD performance using total test time would result in a significant relationship between functional movement, linear speed, jumping ability and COD performance. However, using the CODD as a “gold-standard” method for assessing COD performance, the observed relationship between the analysed performance variables and COD might diminish.

## Methods

### Participants

Fifty highly trained, national level female basketball players (age: 23.7 ± 3.81 years, body height: 175.5 ± 7.69 cm; body mass: 64.4 ± 7.88 kg) competing at the same level in the Spanish N1 Female League were recruited to voluntarily partake in this study. The sample size was calculated using G*Power software (version 3.1.9.6, Kiel, Germany). The number of participants to be included in the study was calculated based on the statistical method used to identify the differences between groups (independent *t*-test). This calculation was based on a large effect size (f) of 0.8, an alpha level of 0.05, and power value of 0.80 (Faul et al., 2007). Inclusion criteria for participants encompassed a minimum of 6 months without lower limb injury prior to the assessment, consistent training of at least 3 days a week throughout the season, in addition to participation in competitive games, and a minimum of 10 years of basketball playing experience. All participants were thoroughly briefed of the possible risks and benefits of study participation, and before the beginning of testing, they provided written consent. This research was approved by the Andalusian Biomedical Research Ethics Committee (protocol code FBD_UHU2020; approval date: 08 October 2020) in adherence to the principles outlined in the Declaration of Helsinki.

### Design and Procedures

A cross-sectional experimental design was employed to analyse and compare the relationships between functional movement, linear speed and jumping ability using two distinct methods of measuring COD performance in female basketball players, with consideration given to different cut-off angles. COD tests included a single cut and the shortest possible approach distance, to minimise the influence of other physical qualities on the test (Gonzalo-Skok et al., 2023; Nimphius et al., 2016). Furthermore, COD tests were performed at different angulations as performance in these actions is directly dependent on the cutting angle (Dos’Santos et al., 2018). All players were tested in a trained state because data collection was carried out during the last phase of the competitive season.

Participants underwent the evaluation during two separate testing sessions, with a 48-h interval between them. All assessments took place on a basketball court just before each training session, scheduled between 19:00 and 21:00, under consistent conditions. A familiarization protocol with submaximal attempts of the proposed tests was executed the previous week. Prior to the evaluation sessions, a 10-min warm-up was conducted, commencing with a general activation including light-intensity jogging, a series of dynamic stretching exercises, and several accelerations, followed by specific potentiation exercises. Additionally, participants were instructed to attend the testing sessions with adequate hydration and rest, refraining from high-intensity training in the preceding 24 h. Moreover, they were advised to regulate their caffeine and food intake at least 3 h before each evaluation.

### Measures

The first testing session was dedicated to quantitative movement tests: a weight-bearing dorsiflexion test (WB-DF) and a Y-Balance Test (YBT), as well as vertical jump tests: a unilateral Countermovement Jump (uCMJ) and a unilateral Drop Jump (uDJ). The second session included all horizontal tests: a 10-m sprint and CODs at different angles (a 505 modified test at 45°, 90° and 180°), and a unilateral Triple Hop Test (uTHT). All players performed a total of three attempts of each test with a two-minute rest interval in between. The mean of all attempts for each test was used for further analysis.

#### Weight-Bearing Dorsiflexion Test (WB-DF)

**Figure 1:**
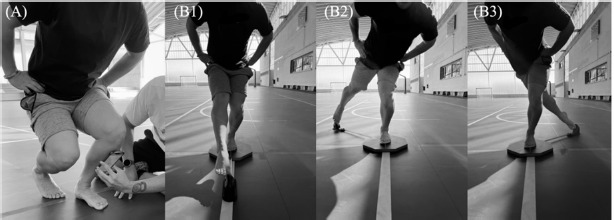
Weight-bearing dorsiflexion test (A) conducted using My ROM App (Apple Inc., Cupertino, CA, USA). Dynamic balance assessment by the Y-balance test (B): anterior reach (B1), posterolateral (B2) and posteromedial (B3) using the OctoBalance device (OctoBalance, Check your Motion, Albacete, Spain).

The WB-DF was carried out with My ROM App (Apple Inc., Cupertino, CA, USA) (Balsalobre-Fernández et al., 2019) by placing the mobile device on the anterior tibial crest, just below the tibial tuberosity, and provided the results in degrees. Each player placed their hands on their hips, as well as the foot to be measured in front and the opposite foot resting just behind. In this position, participants were instructed to lunge forward until their knee reached the maximum range of movement. The heel was required to always remain in contact with the floor (Figure 1A). Players were barefoot for the measurement.

#### Y-Balance Test (YBT)

Dynamic balance was assessed by the YBT using the OctoBalance device (OctoBalance, Check your Motion, Albacete, Spain). While maintaining a balanced position on one foot on the platform, each player had to reach the maximum possible distance in three directions: anterior, posterolateral and posteromedial (Figure 1B). All attempts were supervised by researchers and were considered valid if 1) the heel rested on the back edge of the platform and the second metatarsal was on the front line, 2) the hands were placed on the hips, and 3) the reaching foot only stayed on the platform (Onofrei et al., 2019).

#### Unilateral Countermovement Jump (uCMJ) and unilateral Drop Jump (uDJ)

Jump height in uCMJ and uDJ tests was determined using a Chronojump contact platform (Chronojump BoscoSystem®, Barcelona, Spain) (De Blas et al., 2012). Before testing, participants started with an initial position with one foot on the mat for the uCMJ and from a 25-cm step for the uDJ, then each athlete landed with the same foot on the mat. Athletes were instructed to achieve their maximum jump height with the minimum contact time. The jump was considered valid if 1) the hands were not separated from the hips at any time, 2) the knees were not bent during the flight time, and 3) the athlete landed with only one foot on the same point from which they jumped, holding the position for at least 2 s. In addition, the uDJ was used to calculate the reactive strength index (RSI) of each leg using the flight time/contact time ratio for each jump (Markwick et al., 2015).

#### Triple Hop Test Unilateral

The elastic-reactive force in a horizontal orientation was evaluated through the horizontal triple jump test using a metric tape measure (Hamilton et al., 2008). The test started when the player stood with one leg supported just behind the starting line. After performing three consecutive maximum forward jumps with the same leg, the investigator measured the total distance jumped from the take-off line to the nearest point of landing contact (i.e., back of the heels). Arm swinging was allowed, and attempts were considered failed and thus, then repeated if: 1) the test was not completed as previously described, 2) balance was lost during any part of the test, or 3) the final position could not be maintained on one leg for at least two seconds.

#### Linear (10-m Sprint) and COD (505 Modified at 45º, 90º and 180º) Speed Test

Execution time for speed tests was measured by Chronojump single beam timing cells (Chronojump BoscoSystem®, Barcelona, Spain). The timing cells were placed 2 m from each other with a height of 1.10 m (approximately the height of the players' hips). Before the start of the test, each player was positioned 0.5 m behind the first gate, in a two-point split stance (i.e., starting position with the preferred foot forward and placed exactly 0.5 m behind the starting line). Then, each player accelerated at maximum speed to the second gate located 10 m away for all the tests, in a straight line for the linear test and with a turning point at 5 m where each athlete performed a COD45º, COD90º and COD180º to reach the second gate in the shortest possible time. COD at 45°, 90° and 180° were performed on both sides and laterality was defined by the leg on which participants set on the court when performing the COD mechanics (Cuthbert et al., 2019). The CODD for each angulation (45º, 90º and 180º) was calculated using the formula: ([COD test time – 10-m sprint time] / 10-m sprint time) * 100 (Freitas et al., 2021b); all time variables were reported in seconds.

### Statistical Analysis

The assumption of normality was verified using the Shapiro-Wilk test. Means ± standard deviations (SD) were used to describe variables. The relative and absolute reliability of the tests was evaluated by the intraclass correlation coefficient (ICC) and the coefficient of variation (CV). A median cut-off score was established for each method of determining COD performance and for each COD angulation, thus separating participants according to their performance to each angulation based on the method used. The *High Performance* (HP) group included athletes with a performance above the 50^th^ percentile in each COD test. The *Low Performance* (LP) group consisted of players with a performance below the 50^th^ percentile in each COD test. The Bayesian student's *t*-test for independent samples was used to assess differences between performance groups with regard to both methods, execution time and CODD. Evidence for the alternative hypothesis (H_1_) was set as BF_10_ >1 and evidence for null hypothesis (H_0_) was set as BF10 <1. BF_10_ was reported to indicate the strength of the evidence for each analysis. The BF_10_ was interpreted using the following evidence categories: 1 < BF_10_ < 3 = anecdotal evidence for H_1_; BF_10_ ≥ 3 = moderate; BF_10_ ≥ 10 = strong; BF_10_ ≥ 30 = very strong; BF_10_ ≥ 100 = extreme (Lee and Wagenmakers, 2013). To explore the physical determinants of execution time and CODD, bayesian regression analyses were conducted. R squared was evaluated as < 0.04 trivial, 0.04–0.25 small, 0.25–0.64 moderate, and > 0.64 strong effect (Cohen, 1988). JASP software, version 0.18.1 (Amsterdam, Netherland) for Macintosh, was used for all statistical analyses.

## Results

Table 1 displays mean ± SD values of the assessed variables, along with the ICC and the CV for each. The relative and absolute reliability of the tests was confirmed (ICC ≥ 0.86; CV ≤ 9.89).

The comparison between LP and HP groups in execution time during the COD test for each evaluated performance variable is presented in Table 2. When test execution time served as a measure of COD performance, moderate to extreme evidence supporting differences between groups in vertical jump variables were observed with large effect sizes (ES; ES ≥ 0.66; BF_10_ ≥ 3.50) for all cutting angles. Horizontal variables (sprint and uTHT) exhibited very strong to extreme differences between groups and a larger ES (ES ≥ 0.97; BF_10_ ≥ 44.4) for each angulation, with faster players performing better in all tests. However, no differences were found in functional movement variables between LP and HP groups based on the time of execution of the COD tests. On the other hand, Table 3 outlines differences between each performance variable when CODD was considered as the measure of the COD performance ranking. In that instance, all previously observed differences between performance groups for vertical strength variables disappeared. For CODD45º, moderate evidence supporting differences between groups were found in the linear sprint (BF_10_ = 6.35; ES = 0.72), with players who being more efficient in COD, were slower in linear speed. However, for CODD90° and CODD180°, these differences between groups were found exclusively in the execution time of the COD tests at their respective angles (BF_10_ ≥ 10.1; ES ≥ 0.78), with the most efficient players in COD being the fastest in these actions.

**Table 1:** Descriptive analysis (Mean ± SD) and within session reliability of each performance variable analysed.

Variables	Mean ± SD	Q1	Q3	ICC	CV
**Functional Movement**					
WB-DF R (°)	42.6 ± 5.97	37.8	47.0	0.99	1.08
WB-DF L (°)	42.5 ± 5.64	37.6	46.8	0.98	1.29
YBT R (cm)	62.2 ± 8.76	54.9	68.0	0.92	2.97
YBT L (cm)	62.6 ± 8.70	55.3	68.7	0.89	3.81
**Vertical Force-Vector**					
uCMJ R (cm)	11.1 ± 3.31	9.36	13.3	0.98	5.41
uCMJ L (cm)	11.1 ± 3.47	8.90	12.6	0.88	9.67
uDJ R (cm)	11.0 ± 2.93	8.93	13.7	0.86	9.89
uDJ L (cm)	10.9 ± 3.07	8.88	12.8	0.93	7.12
RSI R	0.68 ± 0.18	0.56	0.78	0.92	8.75
RSI L	0.67 ± 0.17	0.57	0.75	0.95	7.86
**Horizontal Force-Vector**					
uTHT R (m)	4.61 ± 0.54	4.22	5.03	0.93	3.82
uTHT L (m)	4.64 ± 0.59	4.23	4.93	0.93	3.89
10-m sprint (s)	2.01 ± 0.12	1.91	2.09	0.97	1.35
**Change of Direction Test**					
COD45º R (s)	2.14 ± 0.12	2.03	2.21	0.95	1.36
COD45º L (s)	2.13 ± 0.14	2.02	2.23	0.91	3.16
COD90º R (s)	2.42 ± 0.20	2.25	2.55	0.88	3.48
COD90º L (s)	2.42 ± 0.21	2.27	2.57	0.87	3.65
COD180º R (s)	2.95 ± 0.25	2.76	3.14	0.97	1.55
COD180º L (s)	2.95 ± 0.26	2.77	3.11	0.96	1.86

Abbreviations: WB-DF: weight-bearing dorsiflexion; R: right; L: left; º: degree; YBT: Y-balance test including all directions; cm: centimeters; uCMJ: unilateral countermovement jump; uDJ: unilateral drop jump; RSI: reactive strength index; uTHT: unilateral triple hop test; m: meter; s: seconds; COD: change of direction; SD: standard deviation; Q1: quartile 1; Q3: quartile 3; ICC: intraclass correlation coefficient; CV: coefficient of variation

**Table 2:** Differences in performance of quantitative movements and strength in different force-vectors between slow (LP) and fast (HP) basketball players in 505 modified tests at 45º, 90º and 180º.

Variables	LP (n = 25)	HP (n = 25)	Mean Difference (CL 90%)	BF_10_	ES (CI 95%)	Evidence
	Mean ± SD	Mean ± SD				
COD 45º						
Quantitative Movement						
WB-DF (º)	41.1 ± 6.19	43.3 ± 4.89	–2.12 (–4.93; 0.70)	0.56	–0.30 (–0.87; 0.22)	Anecdotal
YBT (cm)	61.2 ± 7.68	63.2 ± 9.57	–1.96 (–6.62; 2.71)	0.37	–0.17 (–0.74; 0.37)	Anecdotal
Vertical Force-Vector						
uCMJ (cm)	9.22 ± 3.09	12.9 ± 2.58	–3.71 (–5.20; –2.22)	152.4	–1.18 (–1.88; –0.49)	**Extreme**
uDJ (cm)	9.41 ± 2.56	12.3 ± 2.78	–2.91 (–4.34; –1.49)	24.1	–0.94 (–1.62; –0.29)	**Strong**
RSI	0.59 ± 0.13	0.72 ± 0.17	–0.13 (–0.21; –0.05)	4.64	–0.71 (–1.36; –0.10)	**Moderate**
Horizontal Force-Vector						
uTHT (m)	4.29 ± 0.37	4.92 ± 0.49	–0.63 (–0.85; –0.41)	923.2	–1.31 (–1.98; –0.64)	**Extreme**
10-m sprint (s)	2.09 ± 0.11	1.93 ± 0.09	0.16 (0.11; 0.21)	4300	1.47 (0.78; 2.16)	**Extreme**
CODD 45º (%)	7.11 ± 4.18	5.38 ± 4.61	1.73 (–0.48; 3.93)	0.59	0.31 (–0.21; 0.88)	Anecdotal
COD 90º						
Quantitative Movement						
WB-DF (º)	42.0 ± 6.32	42.5 ± 5.00	–0.49 (–3.36; 2.38)	0.31	–0.07 (–0.60; 0.46)	Moderate
YBT (cm)	63.3 ± 9.21	61.6 ± 8.49	1.60 (–3.07; 6.27)	0.35	0.14 (–0.40; 0.70)	Anecdotal
Vertical Force-Vector						
uCMJ (cm)	9.35 ± 3.31	1282 ± 2.52	–3.47 (–5.00; –1.94)	58.5	–1.06 (–1.75; –0.39)	**Very Strong**
uDJ (cm)	9.60 ± 2.94	12.2 ± 2.65	–2.58 (–4.05; –1.11)	8.29	–0.79 (–1.45; –0.17)	**Moderate**
RSI	0.59 ± 0.17	0.72 ± 0.14	–0.13 (–0.21; –0.04)	4.05	–0.68 (–1.33; –0.08)	**Moderate**
Horizontal Force-Vector						
uTHT (m)	4.31 ± 0.40	4.90 ± 0.50	–0.59 (–0.82; –0.36)	267.2	–1.17 (–1.84; –0.52)	**Extreme**
10-m sprint (s)	2.08 ± 0.11	1.95 ± 0.02	0.13 (0.07; 0.18)	77.3	1.03 (0.40; 1.68)	**Very Strong**
CODD 90 º (%)	24.6 ± 6.51	16.4 ± 4.69	8.20 (5.36; 11.0)	1088	1.35 (0.66; 2.00)	**Extreme**
COD 180º						
Quantitative Movement						
WB-DF (º)	41.9 ± 6.24	42.5 ± 4.98	–0.59 (–3.45; 2.27)	0.31	–0.08 (–0.62; 0.44)	Moderate
YBT (cm)	61.2 ± 9.61	63.3 ± 8.02	–2.06 (–6.70; 2.58)	0.38	–0.18 (–0.75; 0.36)	Anecdotal
Vertical Force-Vector						
uCMJ (cm)	9.69 ± 3.31	12.7 ± 2.74	–2.99 (–4.59; –1.40)	12.8	–0.85 (–1.52; –0.22)	**Strong**
uDJ (cm)	9.84 ± 2.98	12.1 ± 2.72	–2.25 (–3.75; –0.75)	3.50	–0.66 (–1.30; –0.06)	**Moderate**
RSI	0.66 ± 0.22	0.67 ± 0.10	–0.01 (–0.10; 0.08)	0.31	–0.05 (–0.60; 0.49)	Moderate
Horizontal Force-Vector						
uTHT (m)	4.35 ± 0.44	4.87 ± 0.51	–0.51 (–0.75; –0.27)	38.7	–0.95 (–1.59; –0.33)	**Very Strong**
10-m sprint (s)	2.08 ± 0.12	1.95 ± 0.11	0.12 (0.07; 0.18)	44.4	0.97 (0.34; 1.61)	**Very Strong**
CODD 180º (%)	52.1 ± 7.09	41.5 ± 7.29	10.5 (6.92; 14.1)	1232	1.34 (0.67; 2.02)	**Extreme**

Abbreviations: WB-DF: weight-bearing dorsiflexion; º: degree; YBT: Y-balance test; cm: centimeters; uCMJ: unilateral countermovement jump; uDJ: unilateral drop jump; RSI: reactive strength index; uTHT: unilateral triple hop test; m: meter; s: seconds; CODD: change of direction deficit; COD: change of direction; LP: low performance; HP: high performance; SD: standard deviation; CL: confidence limits; ES: effect size; CI: credible interval. Bold evidences the alternative hypothesis (H1)

**Table 3:** Differences in performance of quantitative movements and strength in different force-vectors between less (LP) and more (HP) efficient basketball players in 505 modified tests at 45º, 90º and 180º.

Variables	LP (n = 25)	HP (n = 25)	Mean Difference (CL 90%)	BF_10_	ES (CI 95%)	Evidence
	Mean ± SD	Mean ± SD				
CODD 45º						
Quantitative Movement						
WB-DF (º)	41.5 ± 5.31	43.0 ± 5.86	–1.57 (–4.41; 1.27)	0.42	–0.22 (–0.78; 0.30)	Anecdotal
YBT (cm)	61.5 ± 7.23	63.1 ± 10.1	–1.64 (–6.28; 3.00)	0.35	–0.14 (–0.70; 0.39)	Anecdotal
Vertical Force-Vector						
uCMJ (cm)	11.1 ± 3.44	11.5 ± 3.32	–0.33 (–2.11; 1.45)	0.32	–0.07 (–0.63; 0.46)	Moderate
uDJ (cm)	11.3 ± 3.03	10.8 ± 3.08	0.52 (–1.09; 2.13)	0.34	0.13 (–0.40; 0.69)	Anecdotal
RSI	0.63 ± 0.14	0.69 ± 0.19	–0.06 (–0.15; 0.03)	0.52	–0.29 (–0.87; 0.26)	Anecdotal
Horizontal Force-Vector						
uTHT (m)	4.59 ± 0.53	4.65 ± 0.56	–0.59 (–0.33; 0.21)	0.31	–0.08 (–0.62; 0.43)	Moderate
10-m sprint (s)	1.96 ± 0.11	2.06 ± 0.13	–0.10 (–0.16; –0.04)	6.35	–0.72 (–1.33; –0.14)	**Moderate**
COD 45º (s)	2.15 ± 0.12	2.12 ± 0.12	0.03 (–0.03; 0.09)	0.39	0.20 (–0.32; 0.74)	Anecdotal
CODD 90º						
Quantitative Movement						
WB-DF (º)	42.4 ± 5.35	42.1 ± 5.91	0.21 (–2.65; 3.08)	0.30	0.03 (–0.50; 0.56)	Moderate
YBT (cm)	62.0 ± 7.86	62.7 ± 6.69	–0.67 (–5.33; 3.99)	0.31	–0.06 (–0.61; 0.48)	Moderate
Vertical Force-Vector						
uCMJ (cm)	10.9 ± 3.11	11.7 ± 3.6	–0.82 (–2.59; 0.94)	0.39	–0.19 (–0.76; 0.35)	Anecdotal
uDJ (cm)	11.4 ± 3.06	10.7 ± 3.03	0.64 (–0.96; 2.25)	0.37	0.16 (–0.37; 0.72)	Anecdotal
RSI	0.65 ± 0.15	0.68 ± 0.18	–0.03 (–0.12; 0.06)	0.36	–0.15 (–0.71; 0.39)	Anecdotal
Horizontal Force-Vector						
uTHT (m)	4.52 ± 0.43	4.71 ± 0.62	–0.19 (–0.46; 0.08)	0.52	–0.28 (–0.84; 0.24)	Anecdotal
10-m sprint (s)	2.00 ± 0.12	2.03 ± 0.13	–0.03 (–0.10; 0.03)	0.40	–0.20 (–0.75; 0.32)	Anecdotal
COD 90º (s)	2.51 ± 0.20	2.34 ± 0.16	0.16 (0.07; 0.26)	10.1	0.78 (0.19; 1.40)	**Strong**
CODD 180º						
Quantitative Movement						
WB-DF (º)	41.7 ± 5.47	42.8 ± 5.79	–1.06 (–3.91; 1.80)	0.35	–0.14 (–0.69; 0.38)	Anecdotal
YBT (cm)	60.7 ± 8.31	64.0 ± 9.06	–3.32 (–7.89; 1.26)	0.55	–0.30 (–0.88; 0.24)	Anecdotal
Vertical Force-Vector						
uCMJ (cm)	10.9 ± 2.95	11.7 ± 3.75	–0.71 (–2.48; 1.06)	0.37	–0.16 (–0.73; 0.37)	Anecdotal
uDJ (cm)	11.2 ± 2.76	10.9 ± 3.36	0.23 (–1.38; 1.84)	0.31	0.06 (–0.48; 0.60)	Moderate
RSI	0.68 ± 0.20	0.65 ± 0.13	0.03 (–0.05; 0.12)	0.36	0.15 (–0.38; 0.72)	Anecdotal
Horizontal Force-Vector						
uTHT (m)	4.47 ± 0.45	4.77 ± 0.59	–0.30 (–0.57; –0.04)	1.33	–0.48 (–1.06; 0.06)	**Anecdotal**
10-m sprint (s)	2.00 ± 0.11	2.02 ± 0.14	–0.02 (–0.09; 0.04)	0.34	–0.13 (–0.67; 0.38)	Moderate
COD 180º (s)	3.07 ± 0.21	2.82 ± 0.22	0.25 (0.14; 0.36)	87.1	1.05 (0.41; 1.70)	**Very Strong**

Abbreviations: WB-DF: weight-bearing dorsiflexion; º: degree; YBT: Y-balance test; cm: centimeters; uCMJ: unilateral countermovement jump; uDJ: unilateral drop jump; RSI: reactive strength index; uTHT: unilateral triple hop test; m: meter; s: seconds; COD: change of direction; CODD: change of direction deficit; LP: low performance; HP: high performance; SD: standard deviation; CL: confidence limits; ES: effect size; CI: credible interval. Bold evidences the alternative hypothesis (H1)

The results of a linear regression analysis, elucidating the variance and properties of each physical variable assessed based on both methods of measuring COD performance are reported in Table 4. All vertical and horizontal strength variables examined showed a significant linear relationship with COD (BF_M_ ≥ 7.897) at every angulation, being linear speed the one that best explained the variance in COD performance (R^2^ ≥ 0.471; BF_M_ > 100) when execution time was taken as a reference measure. Moreover, a one second improvement in linear speed was associated with 0.681, 1.043 and 1.255 s of COD improvement at 45º, 90º and 180º, respectively. This finding contrasts with the relationships found between the strength variables assessed and the CODD. Specifically, only the time in the COD tests at their respective angles showed a significant relationship (R^2^ ≥ 0.409; BF_M_ > 100) with CODD90º and CODD180º, and the time in the linear speed test was related to CODD at 45º (R^2^ = 0.249; BF_M_ ≥ 56.50).

**Table 4:** Bayesian linear regression analysis showing the properties of each physical variable assessed in both COD performance measurement methods.

Independent variable	P (M|data)	Mean (95% CI)	BF_M_	R^2^		P (M|data)	Mean (95% CI)	BF_M_	R^2^
	COD 45º (s)					CODD 45º (%)			
uCMJ	0.995	–0.018 (–0.03; –0.01)	>100	0.319		0.235	0.004 (–0.16; 0.27)	0.307	<0.001
uDJ	0.992	–0.019 (–0.03; –0.01)	>100	0.302		0.314	0.058 (–0.08; 0.48)	0.457	0.025
RSI	0.930	–0.258 (–0.44; 0.00)	13.30	0.206		0.300	–0.910 (–7.81; 2.75)	0.428	0.021
uTHT	1.000	–0.001 (–0.01; –0.00)	>100	0.429		0.236	<0.001 (–0.02; 0.01)	0.308	0.003
10-m sprint	1.000	0.681 (0.51; 0.86)	>100	0.574		0.983	–15.22 (–25.9; –5.13)	56.50	0.249
CODD/COD 45	0.354	0.001 (–0.01; 0.01)	0.549	0.035		0.354	1.993 (–2.68; 12.6)	0.549	0.035
	COD 90º (s)					CODD 90º (%)			
uCMJ	0.995	–0.029 (–0.05; –0.02)	>100	0.318		0.414	–0.162 (–0.79; 0.16)	0.708	0.052
uDJ	0.968	–0.026 (–0.05; –0.01)	29.82	0.243		0.240	–0.021 (–0.45; 0.37)	0.317	0.002
RSI	0.661	–0.207 (–0.60; 0.01)	1.952	0.109		0.282	–1.195 (–11.3; 5.27)	0.393	0.016
uTHT	1.000	–0.002 (–0.01; –0.00)	>100	0.481		0.861	–0.038 (–0.07; 0.00)	6.430	0.157
10-m sprint	1.000	1.043 (0.70; 1.35)	>100	0.501		0.253	–0.984 (–13.8; 5.51)	0.339	0.008
CODD/COD 90	1.000	0.017 (0.01; 0.02)	>100	0.409		1.000	21.09 (13.1; 29.3)	>100	0.409
	COD 180º (s)					CODD 180º (%)			
uCMJ	0.965	–0.030 (–0.05; 0.00)	27.55	0.240		0.306	–0.100 (–0.83; 0.20)	0.442	0.023
uDJ	0.888	–0.026 (–0.048; 0.00)	7.897	0.181		0.235	0.008 (–0.49; 0.58)	0.307	<0.001
RSI	0.321	–0.061 (–0.45; 0.11)	0.472	0.027		0.254	0.898 (–4.75; 13.9)	0.341	0.007
uTHT	1.000	–0.003 (–0.01; –0.00)	>100	0.389		0.600	–0.024 (–0.08; 0.00)	1.499	0.087
10-m sprint	1.000	1.255 (0.81; 1.66)	>100	0.471		0.259	–1.417 (–15.9; 8.64)	0.350	0.010
CODD/COD 180	1.000	0.017 (0.01; 0.02)	>100	0.430		1.000	22.22 (14.4; 31.1)	>100	0.430

Abbreviations: uCMJ: unilateral countermovement jump; uDJ: unilateral drop jump; RSI: reactive strength index; uTHT: unilateral triple hop test; COD: change of direction; CODD: change of direction deficit

## Discussion

Identifying the key factors influencing COD performance is crucial for strength and conditioning coaches who seek to enhance athletes' efficacy in these decisive actions during a basketball game. However, prior to understanding the determinants of COD, it is imperative to analyse the best method for assessing COD performance. Accordingly, the present study undertook an analysis and comparison of the association of functional movement variables, linear velocity and jumping ability considering two distinct methods for measuring COD performance in female basketball players, differentiating between cut-off angles. The main finding of this study is that variables frequently related to COD performance such as dynamic balance (Lockie et al., 2016), linear velocity (Michael et al., 2021; Young et al., 2015), and jumping ability (Barrera-Domínguez et al., 2020; Spiteri et al., 2015), demonstrated a relationship with COD performance when assessed through the traditional method (i.e., execution time in the test). However, this relationship disappeared when COD performance was assessed with a novel approach (i.e., CODD). Furthermore, CODD (90º and 180º) exhibited no relationship with linear speed. Consequently, it would be recommended to use this novel method as the preferred approach for evaluating performance in these actions, effectively isolating the COD capacity from other physical abilities that might influence test outcomes.

Previous research on COD in sport sciences has highlighted the complexity of COD actions which depend on a multitude of technical and physical factors (Sheppard and Young, 2006; Young et al., 2002). However, these findings and conclusions may be due to the way COD has been assessed. Frequently, maneuverability tests have been used. Tests where a variety of movements and COD at different angles are performed, with execution time often employed as the primary metric for assessing the tests results (Nimphius et al., 2018). The execution time in such tests could be influenced by linear speed, anaerobic capacity or the specificity of the movements, potentially masking the player's real ability to perform a COD. In this regard, previous studies (Nimphius et al., 2013, 2016) indicate that the time spent exclusively on COD in these tests is less than 30% of the total test time, allowing athletes with high linear sprinting ability to compensate for potential COD deficiencies (Sayers, 2015). Controlling performance through execution time might not explain a player's COD performance, leading to a need for caution in interpreting previous studies that analysed the determinants of COD performance using the execution time of a maneuverability test as a measure of performance.

In the present study, COD was evaluated using a simplified approach involving a single cut at different angulations. Even with this simplification, significant differences between methods emerged when identifying COD determinants. The results of this study showed a relationship between the traditional method of measuring COD through execution time with the linear speed and jumps regardless of the cutting angle, consistent with findings in previous research (Sheppard and Young, 2006; Young et al., 2002). The RSI showed a strong relationship with COD performance at wide angles, but it was the only variable that showed no relationship with the execution time in COD180º. This may be due to the fact that at these sharper angles, the ground contact times are longer (>400 ms) (McBurnie and Dos’Santos, 2022), with other force variables being more decisive in this instance (Barrera-Domínguez et al., 2024b).

Although COD performance is significantly related to neuromuscular performance as indicated by previous scientific literature (Pereira et al., 2018) and the results so far, when the novel method of measuring COD performance (i.e., CODD) was used as a reference, all previously observed relationships between vertical jump variables and COD disappeared. Large relationships were found between CODD and vertical jump performance in female handball players (Pereira et al., 2018), but in the current study with female basketball players such a relationship between the vertical jump and CODD was not observed. This could be explained due to the sport played by the sample or the different COD tests assessed. Finally, although previous research has found relationships between functional movement variables and COD performance in male basketball players (Gonzalo-Skok et al., 2015), the results of the current study showed no differences between performance groups in any of the COD performance methods examined. Although CODs are complex actions that are executed unilaterally and require adequate lower limb mobility and stability (Gonzalo-Skok et al., 2015), the findings of this study along with previous research (Barrera-Domínguez et al., 2020) may indicate a limited contribution of these functional movement variables to COD performance at any cutting angulation.

On the other hand, previous studies that have used CODD as a metric for assessing COD performance in female samples across different team sports (Freitas et al., 2022; Pereira et al., 2018) have reported significant associations between CODD and linear speed. However, others have not found this relationship between CODD and linear speed (Nimphius et al., 2013, 2016), indicating that CODD is a validated measure which isolates COD from other abilities that may influence the test results and provides more information about the trade-off between linear and multidirectional speed, asymmetries (Barrera-Domínguez, Jones, et al., 2024; DosʼSantos et al., 2019) and the effect of fatigue on these actions (Scanlan et al., 2021). Despite this controversy in the above findings, the current study's results present some additional evidence on the issue, being potentially explained by the specific COD angulation assessed. Performance in COD actions is directly dependent on the angulation of the cut (Dos’Santos et al., 2018). Given the inclusion of different simple COD tests, each featuring a single cut at different angulations in the present study, it was found that CODD at wider angulations exhibited a stronger relationship with linear speed, whereas this relationship was not evident for sharper angles. This, along with the use of different COD assessment tests in previous studies, would explain the abovementioned controversy.

In the context of wider angulations, COD actions should prioritise maintaining maximum velocity while minimizing horizontal braking forces (Bourgeois et al., 2017). This suggests that athletes with higher linear speed could benefit from cutting angulations of less than 60° (Dos’Santos et al., 2018). Conversely, for CODD involving sharper cuts, this relationship with linear velocity disappeared. Such cuts are more force demanding, as the inertia of the movement must be drastically reduced in order to perform the COD efficiently (Bourgeois et al., 2017). Previous studies comparing between genders (Freitas et al., 2021a) have suggested that linearly faster and more powerful male athletes might obtain higher CODD before sharper cuts, given their need to manage greater sprint momentum prior to COD. However, this pattern was not observed in females, as they reached lower linear speeds and, consequently, lower sprint momentums. The present findings align with these observations, revealing no relationship between linear velocity and CODD in a female sample for sharper angulations. Thus, the use of CODD to measure COD performance in female basketball players could be recommended, as it isolates the COD action from other abilities that may influence the tests results.

Before concluding, some limitations should be considered when interpreting the findings of the current study. Firstly, due to the cross-sectional design of the study, a cause-effect relationship cannot be deduced. Secondly, it should be considered that the sample was composed exclusively of amateur female basketball players, thus it is recommended to be cautious when applying the data to other samples. Furthermore, given that we assessed strength variables frequently associated with the execution time in COD tests, these variables did not show large differences or associations with the novel approach. Therefore, it is recommended in future research to analyse other strength and biomechanical variables that may affect the efficiency of COD actions.

## Conclusions

Although a multitude of tests and methods are currently available for assessing COD performance, the findings of this research indicate to practitioners that using CODD to assess this skill through simple COD tests with a single cut at various angles to create an individual “COD angle profile” may be the best way to isolate the COD action. Thus, strength and conditioning coaches could have a complete speed profile for each athlete and analyse the effect of the cut angle on performance of each female basketball player. In addition, it is advisable to exercise caution when considering factors frequently linked to COD performance, given that most prior studies have used test execution time to determine these relationships. As elucidated throughout this study, such relationships are directly dependent on the COD method and angulation assessed, thus generalizations are not advisable. Therefore, coaches and sport scientists are encouraged to use CODD and standardise COD assessments to determine in future research which factors most determine performance in this task.
